# How dispositional optimism relates to psychological resilience: examining the roles of self-efficacy and adaptive coping

**DOI:** 10.3389/fpsyg.2026.1805556

**Published:** 2026-10-16

**Authors:** Zhilin Wang, Hui Hu, Lili Dai, Junhui Wang

**Affiliations:** 1 School of Teacher Education, Lishui University, Lishui, China; 2 School of Education Science, Anqing Normal University, Anqing, China; 3 The Affiliated Hospital of Hangzhou Normal University, Hangzhou, China; 4 School of Early Childhood Education, Hangzhou Polytechnic, Hangzhou, China

**Keywords:** adaptive coping, college students, dispositional optimism, psychological resilience, self-efficacy, sequential mediation

## Abstract

**Background:**

College students face mounting psychological pressures, making the relationship between dispositional optimism and psychological resilience a critical research focus. However, the underlying mechanisms connecting these constructs remain insufficiently explored.

**Purpose:**

This study examines the associations among dispositional optimism, self-efficacy, and adaptive coping strategies in relation to psychological resilience, while controlling for common method variance (CMV).

**Methods:**

We surveyed 504 university students in Zhejiang Province using validated scales. Data were analyzed via correlation analysis, confirmatory factor analysis (DWLS estimation) with ULMC-based CMV assessment, and bootstrapped mediation tests (5,000 resamples) in JASP 0.96.

**Results:**

After controlling for CMV, dispositional optimism showed no significant direct association with psychological resilience (β = 0.192, *p* > 0.05). The indirect pathway via adaptive coping remained significant (effect = 0.030, 95% BootCI [0.004, 0.061]), whereas the indirect pathway via self-efficacy (effect = 0.065, 95% BootCI [−0.016, 0.239]) and the serial mediation (optimism to self-efficacy to adaptive coping to resilience) (effect = 0.014, 95% BootCI [−0.006, 0.069]) were not supported.

**Conclusion:**

The correlational findings suggest that adaptive coping serves as a more stable indirect mechanism linking dispositional optimism to psychological resilience, whereas the previously emphasized roles of self-efficacy and the sequential chain may be inflated by common method variance. Pending replication in longitudinal or experimental designs, interventions could consider directly teaching adaptive coping strategies, rather than assuming a mandatory “self-efficacy first” sequence. This study highlights the importance of controlling for CMV in self-report mediation research and offers a cautious, evidence-informed direction for future resilience-focused interventions.

## 1 Introduction

The contemporary higher education landscape presents university students with multifaceted challenges: academic pressures, career competition, and interpersonal complexities, making mental health a global concern for educators and researchers alike. As a cornerstone construct in positive psychology, dispositional optimism, defined as individuals' generalized expectancy for positive outcomes (Scheier and Carver, 1985), represents not merely an affective trait but a critical psychological resource influencing adaptation and development. Empirical evidence consistently demonstrates optimism's predictive validity for improved work performance (Bai et al., 2025), enhanced engagement (Nagoji and Mackasare, 2023), and academic achievement (Ge et al., 2025).

Conceptually, dispositional optimism comprises cognitive, affective, and motivational dimensions. Cognitively, it manifests as positive outcome expectancies; affectively, it manifests as sustained positive emotionality; and motivationally, it manifests as persistent goal pursuit (Carver and Scheier, 2014). It is posited that optimistic dispositions expand individuals' attentional scope, cognitive flexibility, and behavioral repertoires while cumulatively building enduring personal resources, as described by the Broaden-and-Build Theory (Fredrickson, 2004). Specifically, its broadening function facilitates creative problem-solving in uncertain situations, whereas its building function fosters psychological capital (e.g., self-efficacy, resilience), social capital (support networks), and physiological capital (e.g., cardiovascular health).

Recent empirical work has demonstrated that optimism not only correlates positively with a broad spectrum of physical and mental health indicators (Scheier et al., 2021; Rogowska et al., 2021), but also serves as a critical protective factor that enables individuals to maintain and even enhance psychological resilience during adversity (Huynh et al., 2024). However, the precise mechanisms through which optimism exerts its beneficial effects on resilience remain insufficiently systematized in the literature. From a theoretical perspective, elucidating the intrinsic mechanisms linking optimism with other positive psychological resources would significantly advance personality development theories within positive psychology. At the practical level, a clearer understanding of optimism's operational pathways could provide scientific foundations for developing targeted mental health interventions for university students. This need assumes particular urgency in the post-pandemic era, where heightened societal uncertainties have created unprecedented adaptive challenges for the student population, making the cultivation of optimism especially consequential for resilience enhancement.

Within mental health research, two core constructs have attracted particular scholarly attention: self-efficacy, i.e., individuals' beliefs in their capability to execute goal-directed actions (Bandura, 1997), and adaptive coping strategies, i.e., constructive strategies for managing stressors (Folkman and Moskowitz, 2004). To integrate the three theoretical frameworks in a non-parallel manner, we propose a hierarchical “resource-capability-action” chain. Specifically, drawing on the Broaden-and-Build Theory (Fredrickson, 2004), dispositional optimism serves as an initial psychological resource that broadens cognitive and motivational repertoires. According to Social Cognitive Theory (Bandura, 1997), this broadened mindset then enhances self-efficacy, a cognitive capability judgment that precedes and enables behavioral actions. Finally, Conservation of Resources Theory (Hobfoll, 1989) suggests that increased self-efficacy helps individuals accumulate and deploy adaptive coping behaviors (actions), which directly produce psychological resilience (outcome). These two variables emerge as potential mediators in the optimism-resilience relationship and may serve as critical links between dispositional optimism and psychological resilience, collectively forming a chained mediation pathway: “dispositional optimism to self-efficacy to adaptive coping to psychological resilience”.

The sequential order (optimism to self-efficacy to adaptive coping to resilience) is theoretically non-interchangeable for two reasons. First, self-efficacy represents a cognitive belief about one's capabilities, whereas adaptive coping refers to concrete behavioral strategies. Social Cognitive Theory explicitly posits that efficacy beliefs are proximal determinants of action; individuals must first believe they can succeed before they consistently engage in effective coping behaviors. Second, alternative model structures, such as parallel mediation (self-efficacy and coping operating independently) or reversed order (coping to self-efficacy), contradict this cognitive-behavioral hierarchy. Parallel models would imply that coping can be effective without prior confidence, which contradicts evidence that low self-efficacy undermines coping persistence. Reversed models would suggest that behavioral success precedes efficacy beliefs, which is not supported by the theory's temporal precedence assumptions. Therefore, the proposed sequence is not interchangeable; the cognitive-to-behavioral ordering is theoretically mandated and will be empirically tested against alternative structures.

This integrated framework advances beyond existing mediation studies in three ways. First, prior research often tested isolated mediators (e.g., self-efficacy alone or coping alone) without specifying their sequential relationship. Our model provides a more complete psychological pathway from dispositional optimism to resilience by explicitly ordering cognitive and behavioral components. Second, unlike simple parallel mediation models, our sequential specification allows testing of whether self-efficacy serves as a necessary cognitive precondition for effective coping, a hypothesis that cannot be tested in parallel models. Third, by rooting the sequence in three complementary theories (broadening to efficacy belief to resource conservation), we offer a cross-theoretically anchored explanation that transcends single-framework accounts.

To extend the existing literature, this study aims to examine the mediating mechanisms linking dispositional optimism to psychological resilience through three complementary lines of inquiry. First, we test a sequential mediation pathway—from optimism to self-efficacy to adaptive coping to resilience—while explicitly accounting for common method variance (CMV), which is often overlooked in prior cross-sectional research. Second, we examine whether optimism's association with resilience operates predominantly through indirect pathways, and whether the widely assumed direct effect holds when CMV is controlled. Third, we compare the relative robustness of self-efficacy vs. adaptive coping as mediating mechanisms, with the goal of identifying which pathway offers a more stable foundation for potential intervention strategies. By integrating these three lines of inquiry, the present study seeks to refine theoretical understanding of optimism–resilience pathways and offer cautious, evidence-informed directions for future research.

## 2 Research hypothesis

### 2.1 Total effect hypothesis (H1): the direct effect of dispositional optimism on psychological resilience

Dispositional optimism, defined as a stable personality trait characterized by generalized positive expectations about future outcomes, has been widely documented to enhance psychological resilience across health, education, and occupational domains (He et al., 2013; Thompson et al., 2018). A meta-analysis encompassing 61 independent samples (N > 220,000) reaffirms a robust positive correlation between optimism and favorable health outcomes (r = 0.26, *p* < 0.001), with an even stronger effect size for the absence of pessimism (r = 0.29) (Scheier et al., 2021). This suggests that while optimism and pessimism share protective variance, they may influence resilience through distinct mechanisms.

Focusing on university students, it has been found among 355 U.S. freshmen that optimism was significantly negatively correlated with academic self-concept stress, time pressure, performance anxiety, and academic alienation (r = −0.21 to −0.33) (Huynh et al., 2024). When both optimism and resilience were included in a regression model, resilience independently predicted “academic self-concept stress” (β = −0.42) more strongly than optimism (β = −0.18), indicating that optimism and resilience are synergistic yet distinct constructs. Cross-sectional evidence from Chinese undergraduates further supports this: optimism levels were positively correlated with Connor–Davidson Resilience Scale (CD-RISC) scores (r = 0.38–0.52; Li and Cao, 2020). Additionally, it has been demonstrated in a longitudinal study of Chinese adolescent athletes that optimism directly predicted increased resilience over 6 months (β = 0.22) (Zhang et al., 2024). Integrating the aforementioned theoretical frameworks and empirical evidence, we propose the following hypothesis:

**H1**: Dispositional optimism has a significant direct positive association with psychological resilience.

### 2.2 Mediation path 1 (H2): the independent mediating role of self-efficacy

Bandura's (1997) social cognitive theory posits self-efficacy, the belief in one's ability to organize and execute actions to achieve goals, as a core mechanism linking personality traits to adaptive outcomes. Recent studies have applied this framework to elucidate how optimism enhances mental health and resilience via self-efficacy. For instance, a parallel mediation model was validated among 405 Turkish K−12 teachers, showing that academic optimism not only directly predicted self-efficacy but also indirectly boosted it through two pathways: (1) fostering professional development and (2) enhancing subjective wellbeing (Mavi et al., 2025). This underscores that teachers' beliefs in their instructional success (academic optimism) strengthen their confidence in handling challenges (self-efficacy) by promoting growth experiences and positive affective states.

Similarly, it was reported in a large-sample SEM study (N = 2,578) that self-efficacy mediated 31.6% of the total effect of optimism on subjective wellbeing (standardized indirect effect = 0.21), with this path remaining stable across genders (Yu and Luo, 2018). Longitudinal evidence further revealed a “resource gain spiral”: self-efficacy at Time 2 predicted increased optimism at Time 3 (β = 0.18), which in turn reinforced self-efficacy at Time 4 (β = 0.12) (Li et al., 2019). Notably, it was recently demonstrated that self-efficacy not only directly predicted resilience (β = 0.096) but also indirectly enhanced it by reducing language anxiety and improving emotion regulation, effects that persisted after controlling for common method bias (Xu and Xu, 2025; Pu et al., 2017). Based on the theoretical and empirical evidence presented above, we propose the following hypothesis:

**H2**: Self-efficacy mediates the positive relationship between dispositional optimism and psychological resilience.

### 2.3 Mediation path 2 (H3): the independent mediating role of adaptive coping

Coping theory distinguishes adaptive coping (e.g., problem-solving, seeking support, cognitive reappraisal) from maladaptive strategies (e.g., avoidance, self-blame) (Lazarus and Folkman, 1984). Extensive research highlights adaptive coping as both a buffer against stress and a mediator between traits and psychological outcomes (Connor-Smith and Flachsbart, 2007; Liu et al., 2023). It was found in a study of gastric cancer patients that adaptive coping did not directly predict resilience but fully mediated its effects via self-efficacy and hope (indirect effect = 81% of total effect), revealing a core mechanism: adaptive coping to positive psychological resources (hope/optimism) to psychological resilience (Wu et al., 2021). This was echoed in a study of 1,262 undergraduates, showing that trait adaptive coping reduced depression by elevating resilience (Dong et al., 2024). It was further synthesized that hope and resilience, as key components of positive psychological capital, mitigate mental health issues in students, with coping strategies playing a pivotal role (Yan et al., 2024). Thus, highly optimistic individuals are more likely to adopt adaptive coping (e.g., problem-focused strategies, emotion regulation), thereby bolstering resilience under stress. Integrating the aforementioned theoretical frameworks and empirical evidence, we propose the following hypothesis:

**H3**: Adaptive coping mediates the positive relationship between dispositional optimism and psychological resilience.

### 2.4 Serial mediation path (H4): the self-efficacy to adaptive coping sequential mechanism

The Conservation of Resources (COR) theory posits that initial psychological resources (e.g., optimism) generate secondary resources (e.g., self-efficacy), which then motivate adaptive behaviors to enhance resilience (Hobfoll, 1989).

This “trait-cognition-behavior-outcome” chain mechanism has garnered empirical support. For example, structural equation modeling was employed in a sample of chronic pain patients, finding that optimism significantly predicted adaptive outcomes through the sequential pathway of “self-efficacy to adaptive coping to life satisfaction” (serial indirect effect = 0.07) (Ramírez-Maestre et al., 2019). Their study clearly validated a three-stage resource gain process: highly optimistic individuals, due to their strong belief in their own capabilities (self-efficacy), were more inclined to adopt problem-focused and emotion-regulating coping strategies, thereby achieving better adaptation outcomes.

Additionally, it has been confirmed that both optimism and self-efficacy significantly and positively influence (academic) resilience, suggesting that integrating these variables into a unified model is theoretically justified (Martono et al., 2022). These studies collectively indicate that the “dispositional optimism to self-efficacy to adaptive coping” pathway is not context-specific but rather a robust, cross-domain resource accumulation mechanism.

Applying these findings to the academic and life stressors faced by university students, we propose that higher optimism levels lead to stronger self-efficacy beliefs, which subsequently facilitate problem-solving, support-seeking, and other adaptive coping strategies, ultimately enhancing psychological resilience in challenging situations. Therefore, this evidence supports our proposition that:

**H4**: Self-efficacy and adaptive coping serially mediate the positive relationship between dispositional optimism and psychological resilience.

Building upon the theoretical and empirical foundations outlined above, we propose a conceptual model (Figure 1) to visually elucidate the hypothesized mechanisms through which dispositional optimism influences psychological resilience among university students.

**Figure 1:**
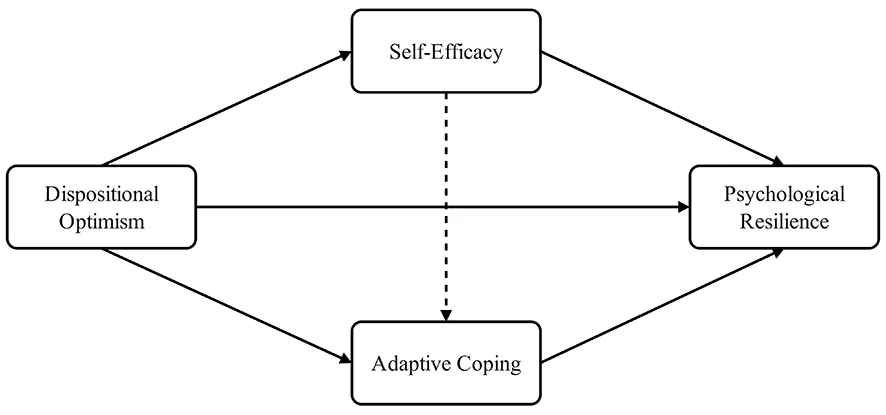
Theoretical framework of the hypothesized chain mediation model. DO, dispositional optimism; SE, self-efficacy; AC, adaptive coping; PR, psychological resilience. Arrows represent hypothesized predictive relationships.

## 3 Research instruments, data, and methods

### 3.1 Research instruments

This study employed a questionnaire survey as the primary data collection method. All scales used were well-validated Chinese adaptations derived from established instruments. During the questionnaire design phase, careful consideration was given to the comprehension level of the target population, ensuring that all items were phrased in clear and straightforward language to obtain accurate responses. The internal consistency reliability (Cronbach's α) for each scale in the current sample is presented in Table 1.

**Table 1:** Theoretical framework of the hypothesized Chain mediation model.

Variables	Scales	Items
Dispositional optimism	LOT-R α = 0.866	In uncertain times, I usually expect the best
		If something can go wrong for me, it will
		I'm always optimistic about my future
		I hardly ever expect things to go my way
		I rarely count on good things happening to me
		Overall, I expect more good things to happen to me than bad
Self-efficacy	GSES α = 0.929	I can always manage to solve difficult problems if I try hard enough
		If someone opposes me, I can find the means and ways to get what I want
		It is easy for me to stick to my aims and accomplish my goals
		I am confident that I could deal efficiently with unexpected events
		Thanks to my resourcefulness, I know how to handle unforeseen situations
		I can solve most problems if I invest the necessary effort
		I can remain calm when facing difficulties because I can rely on my coping abilities
		When I am confronted with a problem, I can usually find several solutions
		If I am in trouble, I can usually think of a solution
		I can usually handle whatever comes my way
Adaptive coping	SCSQ α = 0.862	Engaging in work or study to distract oneself
		Talking to others to vent inner distress
		Trying to focus on the positive aspects of situations
		Reevaluating one's priorities to rediscover what matters in life
		Avoiding catastrophizing (not overestimating the severity of problems)
		Standing firm in one's position and striving for desired outcomes
		Exploring multiple solutions to problems
		Seeking advice from relatives, friends, or classmates
		Modifying one's behavior or addressing personal shortcomings
		Learning from others' approaches to similar challenges
		Making efforts to suppress feelings of disappointment, regret, sorrow, or anger
Psychological resilience	CD-RISC-10 α = 0.922	Able to adapt to change
		Can deal with whatever comes
		Tries to see the humorous side of problems
		Coping with stress can strengthen me
		Tend to bounce back after illness or hardship
		Can achieve goals despite obstacles
		Can stay focused under pressure
		Not easily discouraged by failure
		Thinks of self as strong person
		Can handle unpleasant feelings

LOT-R, life orientation test–revised; GSES, general self-efficacy scale; SCSQ, simplified coping style questionnaire; CD-RISC-10, 10-item Connor–Davidson resilience scale. PSI, person separation index; α, Cronbach's alpha.

#### 3.1.1 Dispositional optimism

Dispositional optimism was measured using the Chinese version of the revised Life Orientation Test (LOT-R; Scheier et al., 1994; Lai and Yue, 2000; Adu et al., 2025), which has been validated in mainland Chinese samples. The scale consists of six scored items: three positively worded (e.g., “In uncertain times, I usually expect the best”) and three negatively worded (e.g., “If something can go wrong for me, it will”). Responses were recorded on a 5-point Likert scale (1 = strongly disagree to 5 = strongly agree), with higher scores indicating greater optimism. In the current sample, the LOT-R demonstrated acceptable internal consistency (Cronbach's α = 0.866).

#### 3.1.2 Self-efficacy

Self-efficacy was assessed using the Chinese version of the General Self-Efficacy Scale (GSES; Zhang and Schwarzer, 1995; Schwarzer et al., 1997), which has shown sound psychometric properties in Chinese populations. The scale comprises 10 items (e.g., “I can always manage to solve difficult problems if I try hard enough”), rated on a 4-point Likert scale (1 = not at all true to 4 = completely true). Higher mean scores reflect stronger self-efficacy. In this study, the GSES exhibited excellent internal consistency (Cronbach's α = 0.929).

#### 3.1.3 Adaptive coping

Adaptive coping strategies were measured using the Simplified Coping Style Questionnaire (SCSQ; Wang and Zhang, 2014), a Chinese adaptation that has demonstrated robust factor structure and good validity. The scale includes 11 items focusing on problem-focused and emotion-regulation strategies (e.g., “Talking to others to vent inner distress”). Responses were recorded on a 4-point frequency scale (0 = never to 3 = often), with higher scores indicating greater use of adaptive coping. In the current sample, the SCSQ showed good internal consistency (Cronbach's α = 0.862).

#### 3.1.4 Psychological resilience

Psychological resilience was evaluated using the 10-item Connor–Davidson Resilience Scale (CD-RISC-10; Campbell-Sills and Stein, 2007; Ni et al., 2016; Connor and Davidson, 2003), a Chinese version validated in Chinese adolescents and young adults. The scale (e.g., “Able to adapt to change”) uses a 5-point Likert scale (0 = never to 4 = always), with higher scores indicating greater resilience. In this study, the CD-RISC-10 exhibited excellent internal consistency (Cronbach's α = 0.922).

### 3.2 Data source and collection procedures

The study sample consisted of 504 undergraduate students recruited from four universities in Zhejiang Province, China. Data collection was conducted through the Wenjuanxing online survey platform (www.wjx.cn) between March 23 and June 16, 2025. A total of 543 questionnaires were initially distributed, with 543 responses received. After removing 39 invalid responses due to duplicate submissions or implausible completion times, we retained 504 valid responses, yielding an effective response rate of 92.8%.

Regarding the sample, faculty collaborators at each institution facilitated voluntary participant recruitment, with all respondents receiving ¥10 RMB compensation upon completion. The final sample size of 504 participants responding to 37 questionnaire items resulted in a participant-to-item ratio of 13.6: 1, substantially exceeding Comrey and Lee's (1992) recommended minimum ratio of 5-10 participants per item and satisfying MacCallum et al.'s (1999) factor analysis requirements.

### 3.3 Data analysis strategy

JASP 0.96 was used for statistical analysis, with all significance tests two-tailed and α = 0.05.

#### 3.3.1 Measurement model modification and validation

In the initial confirmatory factor analysis (CFA), we encountered a Heywood case (i.e., negative residual variance for an observed variable). Examination revealed two items with extreme factor loadings (Q6_Row2 had a very low loading, λ = 0.211; Q6_Row3 had a very high loading, λ = 1.902). After deleting these two items, all data were re-analyzed. We then conducted CFA in JASP 0.96 using the DWLS estimator, which is suitable for categorical variables, to test the four-factor model (optimism, psychological resilience, self-efficacy, adaptive coping). Model fit was evaluated using χ^2^/df, CFI, TLI, RMSEA, and SRMR. Convergent validity was assessed via composite reliability (CR) and average variance extracted (AVE), and discriminant validity was assessed via the heterotrait-monotrait (HTMT) ratio of correlations. As a supplement, we also compared the fit of the four-factor model against a series of alternative models (collapsing some constructs) to further confirm construct distinctiveness.

#### 3.3.2 Common method bias test (ULMC)

To address potential common method bias, we used the unmeasured latent method factor (ULMC) technique (Podsakoff et al., 2012). Specifically, we added a common method factor (CMV) to the four-trait measurement model, with all indicators loading simultaneously on their respective trait factor and on the CMV factor (with the CMV factor constrained to be uncorrelated with the trait factors). Model fit improvement was evaluated using ΔCFI, with ΔCFI > 0.01 indicating the presence of common method variance (Cheung and Rensvold, 2002). If common method bias exists and is severe, the CMV factor will be included as a covariate in subsequent structural equation models to partially eliminate the influence of method variance on the model.

#### 3.3.3 Mediation analysis

First, descriptive analyses were performed to examine the distribution of each variable. Bivariate relationships among study variables were assessed using Pearson correlation coefficients, with effect sizes interpreted according to the recommendations of (Cohen 1988) and (Hemphill 2003) (r ≈ 0.10 = weak, r ≈ 0.30 = moderate, r ≥ 0.50 = strong).

Structural equation modeling (SEM) was then conducted in JASP 0.96 to test both direct and indirect effects (Lei and Wu, 2007) in the hypothesized mediation model (optimism as the independent variable, self-efficacy and adaptive coping as serial mediators, and psychological resilience as the dependent variable).

If the ULMC test indicated no severe common method bias (ΔCFI ≤ 0.01), the baseline four-factor measurement model without a common method factor was used. If severe bias was present (ΔCFI > 0.01), the common method factor (CMV) was included as a covariate in the SEM, with all indicators loading simultaneously on their respective trait factor and on the CMV factor (uncorrelated with trait factors).

The significance of mediation effects was tested using bootstrapping procedures with 5,000 resamples (Hayes, 2018; Hair et al., 2012; Baron and Kenny, 1986) to generate bias-corrected 95% confidence intervals; effects were considered statistically significant when the interval excluded zero. Total, direct, and indirect effects are reported with standardized coefficients (β), along with the proportion of the total effect mediated. All continuous variables were mean-centered prior to analysis. Missing data were minimal ( ≤ 0.5% across items) and handled by listwise deletion; preliminary analyses showed negligible effects of demographic variables on the key variables, so no covariates were included beyond the possible CMV factor.

## 4 Results

### 4.1 Validation of the measurement model

#### 4.1.1 Common method bias

To test for common method bias, the CFA method-factor approach was adopted. First, a measurement model containing only the four trait factors was constructed. Then, a common method factor was added to this model, with all items loading simultaneously on their respective trait factor and the common method factor. As shown in Table 2, the trait-only model had a fit of CFI = 0.889, TLI = 0.881, RMSEA = 0.064, SRMR = 0.056, and χ^2^/df = 3.069. After adding the method factor, the model fit improved to CFI = 0.911, TLI = 0.898, RMSEA = 0.059, SRMR = 0.043, and χ^2^/df = 2.774. Compared with the trait-only model, the addition of the method factor increased CFI by 0.022 and TLI by 0.017, while RMSEA and SRMR decreased by 0.005 and 0.013, respectively. Since ΔCFI exceeded 0.010, the common method factor substantially improved model fit, suggesting the presence of some common method variance in the data. Therefore, the CMV factor was subsequently included in the structural model to test the robustness of the study's conclusions.

**Table 2:** CFA-based common method bias test.

Model	CFI	TLI	RMSEA	SRMR	χ^2^/df
Trait only model	0.889	0.881	0.064	0.056	3.069
Trait + method factor model	0.911	0.898	0.059	0.043	2.774
Change	+0.022	+0.017	−0.005	−0.013	−0.295

CFI, comparative fit index; TLI, Tucker–Lewis index; RMSEA, root mean square error of approximation; SRMR, standardized root mean square residual; χ^2^/df, chi-square to degrees of freedom ratio; “Trait only model”, measurement model with trait factors only; “Trait + method factor model”, measurement model with an uncorrelated latent common method factor added.

#### 4.1.2 Confirmatory factor analysis

**Discriminant validity testing in confirmatory factor analysis adopted conventional thresholds to evaluate model fit:** CFI and TLI ≥ 0.90 indicate acceptable fit (Hu and Bentler, 1999); RMSEA ≤ 0.08 suggests reasonable fit (MacCallum et al., 1996), with values < 0.06 indicating good fit (Hu and Bentler, 1999); SRMR ≤ 0.08 indicates good fit (Hu and Bentler, 1999); and GFI/AGFI ≥ 0.80 are considered acceptable (Sica and Ghisi, 2007). As shown in Table 3, the four-factor model (DO, SE, PR, AC) demonstrated significantly better fit than the three-factor model (combining SE and PR) and the one-factor model (all items loading on a general positive functioning factor). The one-factor model showed poor overall fit (CFI = 0.694, TLI = 0.675, RMSEA = 0.106), indicating that all items cannot be adequately explained by a single general positive functioning factor. In comparison, although the fit of the four-factor model did not reach the ideal level (e.g., CFI = 0.889 < 0.90, RMSEA = 0.064), its fit was clearly superior to the competing models on all key indices, supporting the modeling of DO, SE, PR, and AC as distinct psychological constructs.

**Table 3:** CFA-based comparison of competing measurement models for discriminant validity.

Model	χ^2^/df	CFI	TLI	RMSEA	SRMR	GFI	AGFI
Four factor model: DO, SE, PR, AC	3.069	0.889	0.881	0.064	0.056	0.831	0.807
Three factor model: SE and PR combined	4.157	0.829	0.818	0.079	0.064	0.738	0.704
One factor model:	6.627	0.694	0.675	0.106	0.091	0.618	0.570

χ^2^/df, chi-square to degrees of freedom ratio; CFI, comparative fit index; TLI, Tucker–Lewis index; RMSEA, root mean square error of approximation; SRMR, standardized root mean square residual; GFI, goodness-of-fit index; AGFI, adjusted goodness-of-fit index.

**Convergent and Discriminant Validity**: Using conventional criteria (CR > 0.70 for reliability, AVE > 0.50 for convergent validity, HTMT < 0.85 for discriminant validity; Fornell and Larcker, 1981; Henseler et al., 2015), Table 4 shows that dispositional optimism (DO), self-efficacy (SE), and psychological resilience (PR) demonstrated good convergent validity (AVE = 0.624, 0.574, 0.551, respectively) and composite reliability (CR = 0.869, 0.930, 0.924). Their factor loadings were high (0.579–0.855). Adaptive coping (AC) had acceptable CR (0.864) and discriminant validity (HTMT with SE = 0.714, with PR = 0.701, both < 0.85), but its AVE (0.371) was below 0.50, indicating limited convergent validity, and some loadings were low (0.479–0.701). All HTMT values among the four constructs were < 0.85. Overall, the four constructs are sufficiently distinct for subsequent analyses.

**Table 4:** Summary of factor loadings, AVE, CR, and HTMT correlations.

Construct	Loading range	AVE	CR	1	2	3	4
1. DO	0.756–0.855	0.624	0.869	-			
2. SE	0.636–0.821	0.574	0.930	0.193	-		
3. PR	0.579–0.805	0.551	0.924	0.283	0.800	-	
4. AC	0.479–0.701	0.371	0.864	0.321	0.714	0.701	-

AVE, average variance extracted; CR, composite reliability; HTMT, heterotrait–monotrait ratio.

### 4.2 Correlation analysis among variables

The results showed significant positive correlations among dispositional optimism, self-efficacy, adaptive coping, and psychological resilience (see Table 5). The strongest correlation was between self-efficacy and psychological resilience (r = 0.737, *p* < 0.01), indicating that higher self-efficacy was associated with higher levels of psychological resilience. Self-efficacy also showed a strong positive correlation with adaptive coping (r = 0.636, *p* < 0.01), suggesting that individuals with higher self-efficacy were more likely to adopt adaptive coping strategies. Dispositional optimism was significantly but relatively weakly correlated with self-efficacy, adaptive coping, and psychological resilience, indicating that while dispositional optimism is related to these positive psychological resources, it does not substantially overlap with them.

**Table 5:** Correlations among DO, SE, AC, and PR.

Variable	M	SD	DO	SE	AC	PR
Dispositional optimism (DO)	3.890	0.663	1			
Self efficacy (SE)	2.720	0.578	0.115^**^	1		
Adaptive coping (AC)	2.980	0.473	0.263^**^	0.636^**^	1	
Psychological resilience (PR)	3.565	0.657	0.230^**^	0.737^**^	0.622^**^	1

DO, dispositional optimism; SE, self-efficacy; AC, adaptive coping; PR, psychological resilience.

^**^p < 0.01.

### 4.3 Multiple mediation analysis

#### 4.3.1 Structural paths

As shown in Table 6, without controlling for CMV, all six structural paths in the model were significant, indicating significant positive relationships among dispositional optimism, self-efficacy, adaptive coping, and psychological resilience. After controlling for CMV, only two paths remained significant: the effect of dispositional optimism on adaptive coping (B = 0.081, β = 0.389, *p* = 0.007) and the effect of adaptive coping on psychological resilience (B = 0.364, β = 0.267, *p* = 0.006). In contrast, the direct effects of dispositional optimism on self-efficacy, self-efficacy on adaptive coping, self-efficacy on psychological resilience, and dispositional optimism on psychological resilience were all non-significant (*p* > 0.05). These results suggest that common method variance may have inflated some of the relationships among the variables. After controlling for CMV, the relatively robust structural relationships are primarily reflected in the pathway from dispositional optimism through adaptive coping to psychological resilience.

**Table 6:** Comparison of structural paths before and after controlling for CMV.

Model	Path	B	BootSE	Z	*P*	β
Without CMV	DO to SE	0.097	0.035	2.838	0.006	0.157
	DO to AC	0.124	0.023	5.296	< 0.001	0.218
	SE to AC	0.627	0.066	9.285	< 0.001	0.683
	DO to PR	0.093	0.029	3.182	0.001	0.116
	SE to PR	0.794	0.086	9.409	< 0.001	0.610
	AC to PR	0.339	0.091	3.765	< 0.001	0.239
With CMV	DO to SE	0.146	0.091	1.605	0.109	0.738
	DO to AC	0.081	0.030	2.695	0.007	0.389
	SE to AC	0.269	0.299	0.900	0.368	0.255
	DO to PR	0.055	0.040	1.361	0.173	0.192
	SE to PR	0.443	0.345	1.282	0.200	0.308
	AC to PR	0.364	0.132	2.769	0.006	0.267

CMV, common method variance; DO, dispositional optimism; SE, self-efficacy; AC, adaptive coping; PR, psychological resilience; B, unstandardized coefficient, β, standardized coefficient; BootSE, bootstrap standard error.

#### 4.3.2 Mediation effect testing

As shown in Table 7, without controlling for CMV, the total effect of dispositional optimism on psychological resilience was significant, with an effect size of 0.233 (95% BootCI [0.148, 0.316]); the direct effect was also significant (effect = 0.093, 95% BootCI [0.038, 0.154]). All three indirect paths were significant: the indirect effect of dispositional optimism on psychological resilience via self-efficacy was 0.077 (95% BootCI [0.024, 0.130]); the indirect effect via adaptive coping was 0.042 (95% BootCI [0.019, 0.075]); and the serial mediation effect via both self-efficacy and adaptive coping was 0.021 (95% BootCI [0.006, 0.039]). Thus, without controlling for common method variance, the mediating roles of self-efficacy, adaptive coping, and their serial mediation were all supported.

**Table 7:** Comparison of mediation effects before and after controlling for CMV.

Pathway	Without CMV			With CMV		
	**Effect**	**95% CI**	**Proportion**	**Effect**	**95% CI**	**Proportion**
Total effect	0.233	[0.148, 0.316]	100%	0.164	[0.050, 0.350]	100%
Direct effect (DOtoPR)	0.093	[0.038, 0.154]	39.9%	0.055	[−0.031, 0.127]	33.5%
Total indirect	0.139	[0.066, 0.212]	59.7%	0.109	[0.005, 0.313]	66.5%
Ind1: DOtoSEtoPR	0.077	[0.024, 0.130]	33.0%	0.065	[−0.016, 0.239]	39.6%
Ind2: DOtoACtoPR	0.042	[0.019, 0.075]	18.0%	0.030	[0.004, 0.061]	18.3%
Ind3: DOtoSEtoACtoPR	0.021	[0.006, 0.039]	9.0%	0.014	[−0.006, 0.069]	8.5%

CMV, common method variance; DO, dispositional optimism; SE, self-efficacy; AC, adaptive coping; PR, psychological resilience; BootCI, bias-corrected bootstrap confidence interval.

After controlling for CMV, the total effect of dispositional optimism on psychological resilience remained significant (effect = 0.164, 95% BootCI [0.050, 0.350]), but the direct effect became non-significant (effect = 0.055, 95% BootCI [−0.031, 0.127]). The total indirect effect was still significant (effect = 0.109, 95% BootCI [0.005, 0.313]). However, the specific indirect paths changed: the indirect effect via adaptive coping remained significant (effect = 0.030, 95% BootCI [0.004, 0.061]), whereas the indirect effect via self-efficacy (95% BootCI [−0.016, 0.239]) and the serial mediation effect (95% BootCI [−0.006, 0.069]) were no longer significant. Therefore, after controlling for common method variance, the previously significant serial mediation was no longer robust, and the relatively stable mediation mechanism is mainly the path “dispositional optimism to adaptive coping to psychological resilience.”

## 5 Discussions and implications

### 5.1 Summary of mediation findings and the role of common method variance

Before discussing specific pathways, we first summarize how common method variance (CMV) affected the mediation model as a whole. In the uncorrected model (without a CMV factor), the indirect effects via self-efficacy, adaptive coping, and the serial chain were all statistically significant. After controlling for CMV, two changes occurred: the indirect effects via self-efficacy and the serial mediation became non-significant (95% BootCI [−0.016, 0.239] and [−0.006, 0.069], respectively), whereas the indirect effect via adaptive coping remained significant (effect = 0.030, 95% BootCI [0.004, 0.061]).

This pattern suggests that CMV disproportionately inflated pathways involving self-efficacy, while the adaptive coping pathway was less susceptible to method bias. A possible explanation is that self-efficacy and psychological resilience are both internal, self-evaluative constructs sharing stronger method-related variance (e.g., global self-positivity or common rater effects). Adaptive coping, in contrast, refers to concrete behaviors (e.g., problem-solving and positive reappraisal), which may be less prone to such bias. The substantial changes in significance after adjustment indicate that uncorrected cross-sectional models may produce inflated conclusions. We therefore recommend that future self-report mediation studies routinely test for CMV, either via a latent method factor or through procedural remedies (e.g., temporal separation, anonymity).

### 5.2 The non-significant direct effect

After controlling for CMV, dispositional optimism was not significantly directly associated with psychological resilience (β = 0.192, *p* > 0.05). This pattern is consistent with observations that optimism has a limited direct contribution to psychological adaptation (Huynh et al., 2024), and aligns with the theoretical view that optimism may operate largely through indirect mechanisms. Although early theories emphasized the direct promotive effects of optimism on mental health, the present findings suggest that the association between dispositional optimism and adaptive outcomes predominantly depends on dynamic interactions with other psychological and social resources (Li et al., 2019; Chen et al., 2019; Fu, 2025). Theoretically, these results are also consistent with the TESSERA framework of personality development (Wrzus and Roberts, 2017), which proposes that dispositional optimism may be linked to resilience indirectly via a cascade of adaptive processes (e.g., elevated self-efficacy and the adoption of proactive coping strategies). Compared to prior research (Agbaria and Abu Mokh, 2022), the current study suggests that optimism's association with positive adaptation is largely accounted for by mediation pathways, echoing the argument that optimism does not exert an independent influence on mental health but is embedded within a broader network of psychological resources (Chang, 2024).

### 5.3 Pathway comparisons: self-efficacy, adaptive coping, and serial mediation

#### 5.3.1 The self-efficacy pathway

The non-significant indirect effect via self-efficacy after CMV control raises questions about its role as a stable mediator in the optimism-resilience relationship. Prior studies have suggested that self-efficacy may mediate the link between optimism and wellbeing (Yu and Luo, 2018; Marcionetti and Castelli, 2023). While the present pre-CMV results are consistent with such a possibility, the post-CMV non-significance indicates that the observed mediation may have been partially inflated by shared method variance, such as common rater bias across self-report measures. Cross-cultural research has also shown contextual variability: for example, a reversed pathway (from coach support to resilience to optimism) was observed in athletes (Zhang et al., 2024), and coping flexibility has been associated with self-efficacy and emotion regulation (Zimmer-Gembeck, 2021). These variations suggest that the prominence of self-efficacy as a mediator may depend on sample characteristics, measurement contexts, and the extent of CMV control. Given the cross-sectional design, we cannot determine whether the non-significant pathway reflects a true absence of mediation or an artifact of measurement error and limited statistical power after CMV adjustment. We therefore refrain from claiming that self-efficacy is a robust mediator; instead, the data indicate that other pathways deserve greater empirical priority.

#### 5.3.2 The adaptive coping pathway

In contrast, the indirect effect via adaptive coping remained significant before and after CMV control, indicating that this pathway is relatively robust and less susceptible to common method bias. Theoretically, these findings are consistent with classical theories proposing that optimism promotes adaptive coping strategies, which in turn foster psychological adjustment (Scheier and Carver, 2018). From a social-cognitive perspective (Li et al., 2019), adaptive coping behaviors, such as problem solving and positive reappraisal, appear to directly link optimistic expectations to resilient outcomes, even after accounting for shared variance. Notably, the indirect effect via adaptive coping did not become non-significant when self-efficacy was included as a parallel or serial mediator. This suggests that adaptive coping operates relatively independently of self-efficacy in transmitting the benefits of optimism, contrary to the speculation that coping requires efficacy to be effective. Prior research on nursing students (Shubayr and Dailah, 2025) reported that low self-efficacy may hinder coping effectiveness, but the present correlational data indicate that adaptive coping can mediate optimism–resilience links without necessarily depending on self-efficacy levels. One possible explanation is that the measure of adaptive coping in this study captured general behavioral tendencies rather than situation-specific coping, which may be less reliant on momentary efficacy beliefs. Consistent with a longitudinal study on adolescents with ADHD (Chan et al., 2026), positive expectations and cognitive reappraisal have been shown to enhance resilience through multiple pathways. Our findings complement those results by identifying adaptive coping as a stable indirect pathway, even after rigorous control for method variance.

#### 5.3.3 The serial pathway

The non-significant serial pathway after CMV control indicates that the present data do not provide robust support for the sequential resource-capability-action-outcome gain spiral as originally hypothesized. The proposed sequence from optimism to self-efficacy to adaptive coping to resilience was grounded in social cognitive theory (Bandura, 1997), where cognitive beliefs are expected to precede and enable behavioral actions. While conceptually plausible, the empirical evidence for the full chain is weak after controlling for method artifacts. One possible explanation is that the link between self-efficacy and adaptive coping is less robust than the direct coping-resilience link, or that the two mediators operate in parallel rather than sequentially. This interpretation is consistent with the observation that the adaptive coping pathway remained significant independently, whereas the serial pathway did not. Compared to prior research that observed a reversed pathway (from coach support to resilience to optimism) in athletes (Zhang et al., 2024), the current cross-sectional study does not allow us to confirm or refute the directionality of the serial mediation.

### 5.4 Theoretical and practical implications

These findings refine theoretical understanding of optimism-resilience mechanisms by showing that not all pathways are equally robust under CMV-controlled conditions. The adaptive coping pathway is empirically stable, whereas the self-efficacy and serial pathways appear vulnerable to method effects. This suggests that theoretical accounts should distinguish between methodologically robust mechanisms and those that may be partially inflated by shared variance.

Practically, three implications follow. First, given its stability after CMV control, adaptive coping represents the most directly supported intervention target. Practitioners could implement coping skills training, including problem solving, cognitive reappraisal, and social support seeking, through brief group workshops or counseling services (Khedr and Alshammari, 2025). Second, although self-efficacy became non-significant after CMV control, this does not imply theoretical irrelevance; it suggests that self-efficacy may be more method-sensitive and requires longitudinal replication. Pending such evidence, self-efficacy enhancement (via mastery experiences, vicarious learning, and verbal persuasion) can be considered a secondary or preparatory target, ideally combined with coping training rather than implemented alone. Third, the non-significant serial pathway cautions against a mandatory self-efficacy-first sequence; the data support a parallel pathways approach, allowing practitioners flexibility to prioritize coping directly when time is limited.

## 6 Conclusion

This cross-sectional study examined the associations among dispositional optimism, self-efficacy, adaptive coping, and psychological resilience in a sample of Chinese university students, while explicitly controlling for common method variance (CMV). The results offer three integrated insights.

First, after controlling for CMV, the indirect pathway via adaptive coping remained significant, whereas the direct association, the indirect pathway via self-efficacy, and the serial mediation (from optimism to self-efficacy to adaptive coping to resilience) all became non-significant. This pattern indicates that adaptive coping is the most stable indirect mechanism linking optimism to resilience in the present data, while the self-efficacy-related pathways appear more vulnerable to common method bias.

Second, theoretically, these findings inform the application of social cognitive theory and the broaden-and-build framework to serial mediation models. The lack of robust evidence for the hypothesized sequential chain in the current sample suggests that theoretically assumed sequences may not hold once method artifacts are controlled. Instead, the results align with a simpler, coping-focused pathway, cautioning against untested cascading models without longitudinal or experimental validation. This study thus contributes to the TESSERA framework by demonstrating that the empirical robustness of personality-development sequences depends heavily on methodological rigor.

Third, methodologically, the observed changes in significance after CMV adjustment highlight the importance of controlling for common method variance in self-report, cross-sectional mediation research. Conclusions drawn from uncorrected models may be inflated.

Despite these contributions, several limitations warrant attention in future research. (1) Our sample comprised students from several universities in Zhejiang Province, China. Although the results validated the proposed mechanisms, regional, cultural, and socioeconomic factors may moderate these effects. Future studies should incorporate more diverse samples to examine the generalizability of our findings. (2) This study employed a cross-sectional design, which, while revealing associations between variables, cannot establish causal relationships. Future research could adopt longitudinal tracking or randomized controlled trials to evaluate the practical effects of different interventions, thereby providing more actionable guidance for university mental health education. (3) Given that the current sample may not allow detailed subgroup analyses, future studies could examine measurement invariance of the scales across demographic subgroups (e.g., gender, age, educational level) to further confirm the robustness of the observed patterns.

In sum, despite these limitations, the present study underscores the importance of controlling for CMV in self-report research, suggests adaptive coping as a promising intervention target, and offers cautious, evidence-informed directions for both theory refinement and future investigation.

## Data Availability

The raw data supporting the conclusions of this article will be made available by the authors, without undue reservation.
